# Improving Dementia Awareness in Educators: Evaluating the Impact of a Teacher Training Program

**DOI:** 10.1002/alz70858_104286

**Published:** 2025-12-26

**Authors:** Karen Leticia Pulgatti, Leticia Fernanda Palma, Lea da Silva Veras, Giulianna Bueno Denari, Lucas Nogueira de Carvalho Pelegrini, Marcia Regina Cominetti

**Affiliations:** ^1^ Federal University of São Carlos, São Carlos, São Paulo, Brazil; ^2^ Federal University of São Carlos, São Carlos, SP, Brazil; ^3^ UFSCar, São Carlos, São Paulo, Brazil; ^4^ The Global Brain Health Institute, Trinity College Dublin, Dublin, Dublin, Ireland

## Abstract

**Background:**

Dementia awareness is essential for early identification, enhanced care, and support for individuals living with the condition. Teachers, as essential community figures, can play a significant role in sharing accurate information about dementia. However, limited studies evaluate the impact of dementia‐focused educational interventions on teachers’ knowledge. This study aimed to assess the effectiveness of a teacher training course in improving dementia knowledge using the Dementia Knowledge Assessment Tool 2 (DKAT2).

**Methods:**

The study adhered to all ethical guidelines and was registered under the protocol number CAAE: 58365722.2.0000.5504. A cross‐sectional design was employed, utilizing Google Forms to administer the DKAT2 instrument before and after the intervention. The intervention consisted of a structured teacher formation course on dementia knowledge and awareness. The survey was distributed to primary school teachers across a city in São Paulo state, Brazil. Data were collected anonymously, ensuring participant confidentiality. The DKAT2 responses were analyzed quantitatively to assess changes in dementia knowledge following the intervention. Descriptive and inferential statistical analyses, including paired t‐tests, were conducted to determine significant differences in pre‐ and post‐test scores.

**Results:**

The results demonstrated a significant improvement in dementia knowledge among participating teachers following the intervention. The percentage of correct responses increased across several key areas, including understanding dementia causes, recognizing symptoms, and appropriately responding to individuals living with dementia. Teachers also exhibited greater confidence in supporting students with relatives affected by dementia and in identifying early signs of cognitive decline.

**Conclusion:**

The findings highlight the effectiveness of educational interventions in enhancing teachers' knowledge of dementia. Given their role in the community, equipping educators with accurate dementia‐related knowledge can contribute to early recognition and reduce stigma. Future studies should explore the long‐term retention of knowledge and its application in real‐world settings.

